# Effects of Unconventional Water Agricultural Utilization on the Heavy Metals Accumulation in Typical Black Clay Soil around the Metallic Ore

**DOI:** 10.3390/toxics10080476

**Published:** 2022-08-16

**Authors:** Liang Pei, Chunhui Wang, Liying Sun

**Affiliations:** 1 Xinjiang Institute of Ecology and Geography, Chinese Academy of Sciences, Urumqi 830011, China; 2 Institute of Geographic Sciences and Natural Resources Research, Chinese Academy of Sciences, Beijing 100101, China; 3 University of Chinese Academy of Sciences, Beijing 100049, China

**Keywords:** unconventional water agricultural utilization, cucumbers, heavy metals, soil–crop system, typical soil irrigation area around the metallic ore

## Abstract

Unconventional water is an important water resource for agricultural utilization in the drought and water shortage of Northeast China. Additionally, exploration in making full use of it is an important way to alleviate water shortage in China. This paper analyzed the effects of unconventional water through field trials on the accumulation of heavy metals in both cucumbers and the typical black clay soil (expressed as black soil) around the Anshan metallic ore. By exploring the effects of unconventional water after secondary treatment on the accumulation characteristics of heavy metals in cucumbers and the heavy metal balance in the soil–crop system under different conditions, the study shows that there are no significant differences in the heavy metal content when the quantity of unconventional water for irrigation varies. Unconventional water for short-term irrigation does not cause pollution to either the soil environment or the crops. Nor will it cause the accumulation of heavy metals, and the index for the heavy metal content is far below the critical value of the trade standard and national standard, which indicates that the crops irrigated with unconventional water during their growth turn out to be free of pollutants. Unconventional water brings less heavy metals into the black soil than crops. The input and output quantities have only small effects on the heavy metal balance in the black soil. This paper provides a reference for the safety control and evaluation of unconventional agricultural utilization.

## 1. Introduction

There is a shortage of water resources in northern China. Coordinating the water resource allocation scheme between different water sources and water users can effectively adjust the contradiction between water supply and consumption. Additionally, the scientific allocation of regional water resources can be gradually realized. In view of these problems, the development and utilization of sewage and unconventional water resources has become an inevitable choice and development trend. Similarly, water resources are very important strategic and economic resources. The research on sewage recycling and unconventional water resources utilization technology is an important basis for the scientific allocation of water resources. In 2020, China’s agricultural water consumption accounted for more than 62% of the total annual water consumption. From the perspective of resources, the uneven spatial and temporal accumulation of water resources in China, the poor matching of cultivated land resources and the imperfect engineering facilities system have highlighted the contradiction between supply and demand of water resources in many regions. In the case of water shortage and low utilization rate of agricultural water, there is an urgent need to find alternative water sources and put forward feasible solutions. The development of unconventional water resources and the comprehensive utilization of agriculture have become inevitable choices and important methods. At the same time, the collaborative allocation of unconventional water and conventional water resources is an attempt to seek to coordinate the whole water-resources system [1,2].

Unconventional water includes rainwater, mine water, reclaimed water, salt water, brackish water, etc. The effects of unconventional water on the physical properties of soil change along with characteristics of the irrigated soil. The effects of unconventional water on the chemical properties of soil are mainly reflected in the changes of N, P and saline elements [3,4]. Unconventional water irrigation, which contains a large quantity of nutrient elements, helps to improve soil fertility. Since an unconventional water resource contains traces of saline elements, long-term irrational irrigation will affect soil permeability; specifically, an excessive accumulation of saline elements in plant roots changes the soil composition, resulting in soil compaction. Certain ions of soil’s saline elements have toxicity, and also lead to changes in the soil’s physical environment. An increase in sodium ions reduces the soil porosity, resulting in a decrease in the retention capacity of nutrient elements in soil. The negative and positive effects that an unconventional water resource has on fertility and pollution both determine the effects of sewage irrigation on the physical and chemical properties of soil; in particular, its effects on the degree of soil fertility have become a hot research topic during the process of unconventional water agricultural utilization [5,6,7,8]. However, the complexity of migration and transformation of unconventional water resource in soil system also makes the effects of unconventional water on soil fertility a difficult topic [9,10,11,12].

Northeast China is extremely rich in metallic ore, especially in Anshan and other places, but the economic level is weak, and the treatment and utilization efficiency of water resources is low. The use of unconventional water is still in its infancy [13,14]. For some economic and technical reasons, some pollutants in unconventional water are not completely removed [15]. The N, P, high quantity of completely saline elements, different kinds of toxic traces and substances (heavy metals in black soil irrigation area around the metallic ore, organic pollutants, etc.) as well as pathogens may become new sources of pollutions. Adversely, these harmful substances may have negative effects on soil and crops. Will the heavy metals in the black soil irrigation area around the metallic ore enter the soil with water through unconventional water agricultural utilization, and then be absorbed by crops in the process of crop growth, completing the transfer of water to soil, and then to vegetables? The heavy metals in black soil irrigation area around the metallic ore that have entered into soil and crops will not harm the environment and crops during a limited period of time [16]. When their accumulation overtops the volume that soil and crops can support, they will harm the crops and human beings, and cause serious ecological problems. This can explain the fear some people have of eating local vegetables in Anshan city, since they feel there are heavy metals in the black soil irrigation area around the metallic ore therein. Therefore, it is important to understand the migration and transformation law of heavy metals in soil and crops around the black soil ore area. Both domestic and foreign scholars have conducted research on the unconventional water agricultural utilization [17,18,19,20]. However, most of the research focuses on physiological and biochemical effects on crops, and only few of the studies emphasize the quality, crop fruits and soil metal contents [21,22].

In view of the above problems, we set up an observation and test station in a metal mining area in Anshan city to conduct in-depth study of unconventional water utilization. In this study, the agricultural utilization of unconventional water was studied in the irrigation area around an ore area in Anshan city [23,24,25]. This article, by experimenting with unconventional water agricultural utilization on cucumbers in drip conditions, studied the effects of unconventional water agricultural utilization on heavy metals in cucumbers and soil; explored whether unconventional water, after a secondary treatment, can be used for irrigation and for content and accumulation feature in cucumbers; and analyzed the heavy metals balance of soil–crop system. This paper provides a basis for evaluating the impact of unconventional water agricultural utilization on soil environment and formulating agricultural safety control standards in mining areas and black soil areas. It also provides a scientific and theoretical basis for the scientific allocation of water resources in the black soil region of Northeast China.

## 2. Materials and Methods

### 2.1. Situation of Nature and Crops in the Experimental Site

This study was based on the “Independent research projects of Xinjiang Institute of ecology and geography, Chinese Academy of Sciences” project by the Chinese Academy of Sciences. It was located near a metallic ore area in Anshan city, Northeast China, and belongs to a semi-arid and semi-humid climate area. It has four distinct seasons, including a long winter and a short spring. In spring, the temperature rises rapidly, with more rainfall in autumn and less rainfall and snowfall in winter. The temperature is moderate. The annual solar radiation was 106.6 kcal/cm^2^, the physiological radiation was 50.4 kcal/cm^2^, and the sunshine time was 1629.8 h. The annual average temperature was 13.3 °C, the extreme low temperature was −24.7 °C and the extreme high temperature was 38 °C. The annual accumulated temperature (≥8 °C) was 4396.2 °C. The frost-free period was 213 days per year. The average annual rainfall was 615 mm. The interannual precipitation changed greatly. The precipitation in flood season (from June 1 to July 30) accounted for about 58% to 62% of the whole year. The rainfall intensity was medium, the rainfall duration was short, and the infiltration capacity was limited. Rainwater also easily rubs and erodes the soil surface. In this study, the experimental soil is a unique black soil in Northeast China, with a unit weight of 1.16 to 1.31 g/cm^3^ [4,9].

In total, the test site has 48 test plots, and the area of each plot is 3 m × 3 m. During the test, among the 18 plots of the north section, plots 1–12 and 13–24 were irrigated with clear water and unconventional water, respectively, while all 24 plots of the south section had always been irrigated with clear water before 2018. Between 2016 and 2020, full reclaimed water treatment, rotation irrigation, and mixed irrigation with unconventional water and clean water treatment were set in 13–24 plots of the north section and 24 plots of the south section, respectively. The rotation irrigation adopted an alternative irrigation method in order to effectively take advantage of reclaimed water and clean water. During the test, the amount of irrigation water was 500 m^3^/hm^2^, but the irrigation time and frequency were determined according to the actual situation of the growth period [17,23].

Cucumber plants were selected as the test crop; the unconventional water irrigation test had been carried out in this greenhouse for two years. Cucumber seedlings growing well and uniformly were selected for the test; each of them had three leaves and one core and was 25 cm high. The test ended on 20 September and lasted for three months. Such tomatoes were subject to 35 cm of row spacing, 50 cm of plant spacing and 35,000 plants/hm^2^ of planting density. Sandy clay loam was selected for the test and the soil dry bulk density was 1.3 g/cm^3^. A drop irrigation mode was adopted, and a drop irrigation tube was set for each row of crops, of which the head flow was 1.8 L/h and the irrigation amount was controlled by water meters. The unconventional water used during the test was the effluent from simple constructed wetland, and the clean water was groundwater from the test station [8,11,25].

Table 1 shows the contents of seven heavy metals present within the irrigation water. It can be seen from Table 1 that, besides Hg, the contents of other seven heavy metals in the unconventional water are higher than those in the groundwater from the test station. Among others, the contents of As, Cd, and Cr in the unconventional water are about twice as high as those in the groundwater, 3 times for Hg, 10/8 times for Cu/Zn and one order of magnitude for Pb. However, the contents of heavy metals in unconventional water and groundwater are far lower than the upper limits prescribed in the Agricultural Irrigation Water Quality Standard (GB5084-92).

In the test station, the unconventional water from villages in Anshan mining area is used, which is the secondary treatment unconventional water in a simple constructed wetland. The water quality is stable and can be used at any time.

### 2.2. Monitoring Items and Methods

The soil and cucumber sampling times were July 2019 and September 2020, respectively, which is the time for cucumber harvest. The soil samples were taken from 0~30, 30~60 and 60~90 cm soil layers in each small cell. Test indexes included TN, NH^4+^–N, NO^−^_3_-N, organic nitrogen, available potassium, available phosphorus, Ca^2+^, Mg^2+^, Na^+^, CO^2−^_3_, HCO^3−^, SO^2−^_4_, pH, EC, CEC, As, Cd, Cu, Cr, Hg, Zn and Pb. The sampling time for the crops was September 2020, cucumbers harvest time, and the sample was the fruits. The test indexes were as follows: seed and fruit dry weight, and TN, NO^−^_3_-N, NH^4+^–N, TP, As, Cr, Cd, Hg, Cu, Zn and Pb contents in them [4,7].

### 2.3. Instruments and Reagents

Instruments: The inductively coupled plasma emission spectrograph (ICP-AES), bath water kettle, PHs-11A model Digital display ion meter, electric vibrating machine, analytical balance, electric dry oven, etc.

Reagents: perchloric acid (which is analytically pure), nitric acid (guarantee reagent), hydrofluoric acid (analytically pure), hydrogen peroxide, phenanthroline indicator, 1.60 mol·L^−1^ potassium dichromate standard solution, de-ionized water, 0.42 mol·L^−1^ of ferrous sulphate solution, paraffin vegetable oil and concentrated sulfuric acid.

### 2.4. Samples Determination Method

To determine the contaminants of soil and cucumbers in a black soil irrigation area around the metallic ore contents, we picked cucumbers twice a week during the full productive period. Each measurement was repeated at each production process, taking the average as the final output. We picked 10 ripe cucumbers from each small cell and determined the heavy metals contents of cucumbers. We used the mass spectrometer MDS28 microwave digestion instrument and the inductively coupled plasma ELAN5200 model to determine the heavy metals of cucumbers [26,27,28]. Determination method: we cut cucumbers into pieces, put exactly 0.1200 g into the PTFE digestion pot, added 1 mL of concentrated H_2_O_2_ and HNO_3_ each, used expander for expansion of the packed piston seal, and covered inside the lid. Dry water drops were placed on the outer wall of inner pot, and the inner pot was equipped with pads placed outside the pot, which had a cushion block inside. We put it in the pot shelf and put the gland on the pot shelf along two screws, with the selected Microwave digestion process to digest. We removed the pot after sufficient cooling. The solution was quantitatively transferred to a 10 mL tube. We added 20 mg/mL standard internal mixture set to 10 mL volume. At selected operating parameters ICP-2MS, we determined the digested sample solution Pb and As, and the mass concentration of Hg, Cd and other elements directly.

We determined Pb and Cd in soil using graphite furnace atomic absorption spectrometry, and heavy metals in black soil such as Zn, Cu and other contents in soil using flame atom absorption spectrometry.

All the above large-scale equipment are from Shanghai Instrument Factory, and small-scale instruments were purchased from ordinary stores.

### 2.5. Experimental Data Analysis

We analyzed all the data using the SPSS12 statistical analysis software. Simultaneously, we also used methods of analysis of variance and comparison of multiple groups of continuous variables.

## 3. Results

### 3.1. Heavy Metals Contents of Unconventional Water in Black Soil Irrigation Area around the Metallic Ore

In Table 1, there are seven heavy metals in unconventional water of black soil irrigation area around the metallic ore. As can be seen from Table, the Pb content was similar to that in normal water, while other kinds of heavy metals in unconventional water of black soil irrigation area around the metallic ore were higher than those in normal test water of this experimental area. Among those, the As, Cr, Cd and Hg contents were about twice that of normal water, and Cu and Zn, respectively, had a difference of 10 and 8 times compared to the normal water. However, the heavy metals in unconventional water of black soil irrigation area are much lower than the upper limit of the agricultural utilization water standard (GB5084-08).

### 3.2. Effects of Unconventional Water of Different Periods on the Heavy Metals Content in Black Soil

Table 2 shows the accumulation of six heavy metals in black soil with different unconventional water agricultural utilization periods. As can be seen from the table, the background values (test values in September 2019) were presented, and the soil heavy metals contents with 12 months’ unconventional water agricultural utilization (test values in September 2020 with unconventional water agricultural utilization), with 18 months’ unconventional water agricultural utilization (test values in September 2019 with unconventional water agricultural utilization) and with 24 months’ unconventional water agricultural utilization (test values in September 2019 with unconventional water agricultural utilization) all decreased with the increase in soil depth. This proved that for the period (≤24 months), unconventional water agricultural utilization had no obvious effect on heavy metals accumulation in black soil layers [29,30]. Compared with the soil environmental quality standard (GB15618-2008), even with a large heavy metals content of the surface layer (0~30 cm) of the black soil, the heavy metals contents in black soil irrigation area around the metallic ore were much lower than the upper limit of the target value of the soil environmental quality standard prescribed level, where the contents were the highest. This indicated that unconventional water irrigation in a short term (≤24 months) will not cause heavy metals accumulation pollution in black soil irrigation area around the metallic ore. This result was consistent with the results of Adrien et al. and María et al. [31,32].

As shown in Table 2, by comparing heavy metals contents in 2019 and 2020 with unconventional water agricultural utilization with background values, using multiple comparisons (LSD method) analysis, in which As, Cu, Cr, Zn, Hg contents in 0–30 cm soil layer showed some differences, but no significant differences at other depths for As, Cu, Cr, Zn, Hg and other heavy metals in black soil irrigation area around the metallic ore.

The cause of the above difference was probably the difference in sampling locations in different years. Due to the spatial variation of the black soil properties, the black soil structure, clay particle contents, infiltration capacity and organic matter concentration were not the same in different sampling locations, which resulted in fluctuation and differences in heavy metals contents in black soil in different years. Overall, the seven heavy metals did not accumulate in all the black soil layers with extended years of unconventional water resource irrigation utilization. In addition, other factors such as differences in irrigation and rainfall infiltration space, atmospheric dust, heavy metals in black soil background and other crops uptake, etc., also had some impacts on soil heavy metals content difference to some extent in black soil irrigation area around the metallic ore.

### 3.3. Effects of Different Volumes of Unconventional Water Agricultural Utilization on the Content of Heavy Metals in Black Soil Irrigation Area around the Metallic Ore

Depending on the circumstances of the experimental area, during the period 2019–2020, different water resources were used to irrigate different test plots and according to different requirements. These plots are irrigated by clean water and unconventional water alternately, and full-unconventional water [33,34]. The latter two kinds of water quality were called half-unconventional water and full-unconventional water. The heavy metals contents in black soil irrigation area around the metallic ore were shown in Table 3.

As seen from Table 3, when comparing the value of different volumes of unconventional water irrigation utilization and clear water, there was no significant difference in the most soil heavy metals contents of black soil irrigation area around the metallic ore. For the heavy metals’ impacts on the environment, the unconventional water resource can substitute clear water as the irrigation water source. There was no significant difference in the heavy metals contents between different volumes of unconventional water irrigation except Zn and Cd in 30–60 cm deep soil, which might be caused by soil spatial variability of the soil sampling locations. This indicated that the impact of unconventional water irrigation utilization on black soil heavy metals does not cause heavy metals accumulation in the cucumber growing season.

### 3.4. Accumulation Characteristics of Heavy Metals in Cucumbers under Unconventional Water Agricultural Utilization

The Cr, Cd, Pb, Hg, As, Cu and Zn metals were selected, which have a major effect on the crop growth and human body food chain, and they were analyzed. The analysis result of heavy metals contents in the cucumber fruits was shown in Table 4. Statistical analysis showed that the heavy metals content accumulation had the following characteristics: for irrigation water quality under different conditions, there were different accumulation characteristics of heavy metals in cucumber fruits [35,36]. The Cr, Cd, Pb, Hg, As and Cu contents increased, along with a volume of unconventional water irrigation. Using variance analysis, the variation of Cr content was found to reach significant level of 88% (F = 5.891, sig = 0.079 < 0.1), but for Cd, Pb, Hg, As, Cu and Zn, it did not reach this significant level. Zn existed in relatively small proportions in the cucumber fruit, so it could not be detected from the experiment. Although several kinds of ions showed an increasing trend in the cucumber fruit, they were far below the Safety Qualification for Agricultural Product national standard (GB2763-2012) and safety requirements for pollution-free vegetables quality standard [37,38]. The above analysis and comparison indicated that irrigation via an unconventional water resource was more secure than that using the sewage resource. In addition, compared with clear water for irrigation, unconventional water for irrigation did not cause a significant increase in the heavy metals in cucumber fruits. This further indicated that short-term unconventional water irrigation utilization had little effect on heavy metals in the crops of black soil irrigation area around the metallic ore.

### 3.5. Analysis on Heavy Metals Content in Black Soil and Cucumbers under Unconventional Water Agricultural Utilization

**Figure 1:**
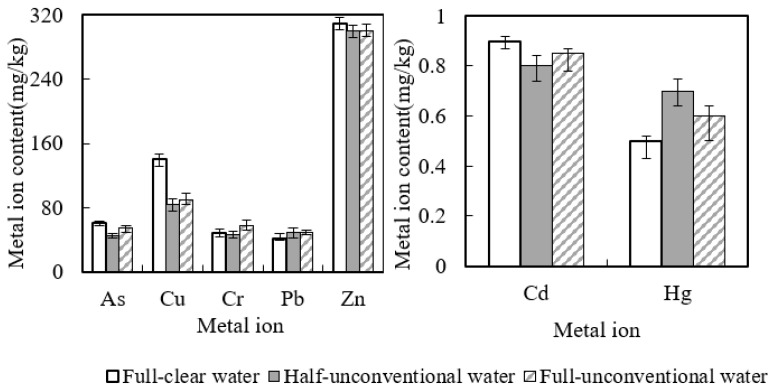
Heavy metals take-away by the aboveground part of cucumber. mg/kg.

**Figure 2:**
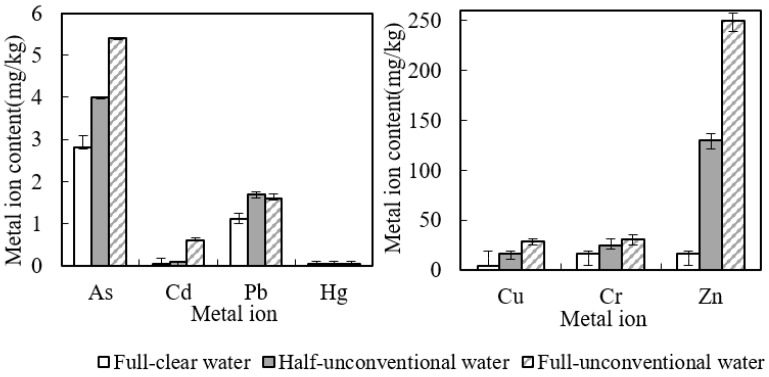
Heavy metals bring-in with the unconventional water for irrigation. mg/kg.

During the cucumber growing season in 2019–2020, according to the heavy metals volume in black soil irrigation area around the metallic ore that entered into the soil with unconventional water irrigation utilization and that was taken away by the aboveground part of the cucumber, this paper analyzed the heavy metals content balance status in black soil and cucumber, as shown in Table 5, Figure 1 and Figure 2. It can be seen from the table that only seven heavy metals were found in the black soil irrigation area around the metal mine. The amount of heavy metals carried by the above ground part of cucumber is higher than that brought by unconventional agricultural water. Additionally, in the full-unconventional water for irrigation condition, the take-away for heavy metals As, Hg and Cd in black soil was, respectively, 10, 8 and 18 times the bring-in. For Pb, take-away was about 24 times the bring-in. Additionally, for Cu, Cr and Zn, it was, respectively, 1.8, 2.3 and 1.8 times, respectively. Taking advantage of the unconventional water for irrigation utilization, analyzing the balance relationship of heavy metals in black soil and crops in condition of the unconventional water for irrigation utilization, it was observed that this water helped to discharge heavy metals, the same as clear water and mixture agricultural irrigation utilization [39,40,41]. This may affect the heavy metals in black soil. However, as we can see from Table 3, the soil heavy metals contents in black soil irrigation area around the metallic ore did not show a significant change (increase or decrease) before and after the cucumbers’ growth season. So, except for the effect of water quality, the difference in take-away and bring-in for the soil heavy metals in black soil irrigation area around the metallic ore contents may settle with precipitation, evaporation, fertilization, atmosphere and other factors.

**Figure 3:**
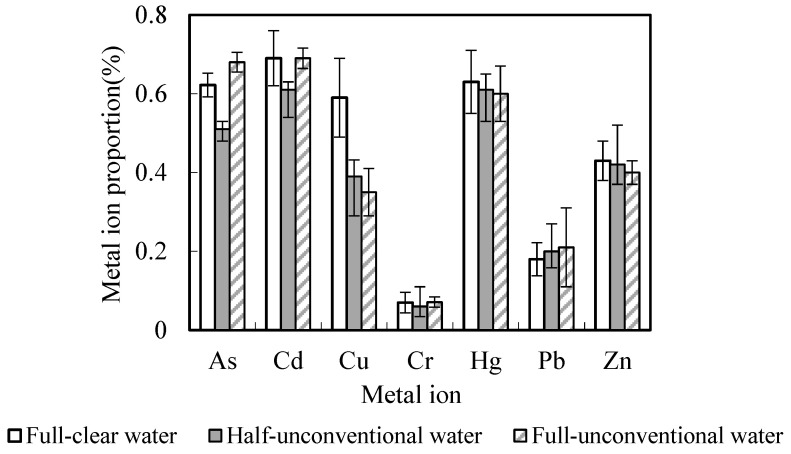
Heavy metals take-away proportions account for total volume in soil. %.

**Figure 4:**
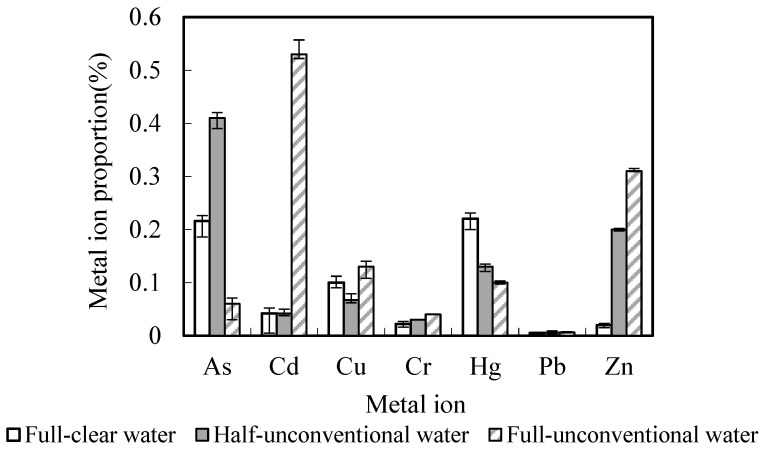
Heavy metals bring-in proportions account for total volume in soil. %.

The proportion of heavy metals take-away and bring-in in the black soil depth of 0 to 90 cm was shown in Table 6, Figure 3 and Figure 4. As had the highest take-away proportion of 0.88~0.96%, while Cr has the lowest, ranging from 0.064~0.092%. Zn accumulated the highest bring-in proportion of 0.09~0.43%, and Pb accounted for the lowest bring-in proportion, 0.0061~0.013%. This showed that both the bring-in and take-away for heavy metals contents accounted for very little proportions of the total heavy metals contents in the black soil depth of 0–90 cm. In conclusion, bring-in with unconventional water irrigation and take-away by the aboveground part of the crop have little effect on the heavy metals contents balance in black soil.

## 4. Conclusions

In order to solve the problems of water shortage and low utilization of unconventional water in Northeast China, a test station was established near a metal mine in Anshan city to carry out research on unconventional water irrigation. It was found that the geochemical behavior of heavy metals in the black soil irrigation area around the metal mine is related to the physical and chemical properties of the soil, the types of vegetables and the characteristics of the elements themselves. The heavy metal content in the black soil and cucumber fruits, volume migration and accumulation rate and element correlation were comprehensively analyzed. They indicated that migration accumulation rate of various heavy metals volume in black soil had different correlation during the heavy metals migration process in the soil and cucumber fruit. The main conclusions were drawn as follows:

(1) Different volumes of unconventional water had no significant effect on the soil heavy metals volumes. For the seven kinds of heavy metals in the black soil irrigation area around the metallic ore, the take-away by the crop harvest was higher than the bring-in when irrigated by unconventional water. Among all the heavy metals in black soil irrigation area around the metallic ore, the take-away volumes were, respectively, 10, 8 and 18 times the bring-in for metals such as As, Hg and Cd. For Pb, the take-away was about 24 times the bring-in, but both the bring-in and take-away accounted for very small proportions of total heavy metals contents of the black soil in depth of 0–90 cm. This showed that the unconventional water agricultural utilization had little effect on the heavy metals pollution in the black soil irrigation area around the metallic ore.
(2) The field experiment using an unconventional water resource to irrigate cucumbers showed that heavy metals increased in the agricultural soil environment, but there was no significant difference. The heavy metals volumes in black soil and crops were far below the food hygiene permission value standards and national soil environmental quality standard. So, the unconventional water agricultural utilization would not cause accumulation of heavy metals pollution to agricultural soil environment and crops.
(3) Unconventional water agricultural utilization is not the only decisive factor for heavy metals volume change in black soil and crops. That is also affected by climate change, fertilization, the soil self-purification capacity, soil and crop types, and similar factors. It is worth further studying the effects of unconventional water agricultural utilization on crop nutritional, crop quality and morphologic change; the relationship between unconventional water quality and the crop yields; and the safety of organic and hazardous substances.

The results provide a basis for evaluating the impact of unconventional water agricultural utilization on soil environment and formulating agricultural safety control standards in mining areas and typical soil areas. It also provides a scientific and theoretical basis for the scientific allocation of water resources in the typical soil region of Northeast China.

## Figures and Tables

**Table 1:** Heavy metals in the unconventional water of black soil irrigation area around the metallic ore.

Water Quality	As /mg·L^−1^	Cr /mg·L^−1^	Cd /μg·L^−1^	Hg /mg·L^−1^	Cu /μg·L^−1^	Pb /mg·L^−1^	Zn /mg·L^−1^
All Clear water (underground water)	0.0026	0.0157	0.031	0.0026	0.029	0.0079	0.0250
Unconventional water	0.0059	0.0343	0.065	0.0062	0.272	0.0081	0.1750
Agricultural utilization water quality standard	0.05	0.1	0.5	1	1	0.1	2

**Table 2:** Soil heavy metal contents for different unconventional water in different irrigation periods and background values.

Index	Depth/cm	Background Values	Irrigation Periods			Soil Quality Standard GB15618-2008		
			12 Months	18 Months	24 Months	First Grade	Second Grade	Third Grade
As /mg·kg^−1^	0~30	8.4 a	9.8 a	6.7 a	9.5 b	≤15 ≤ 25 ≤ 40		
	30~60	8.1 ab	9.0 a	6.1 b	8.4 a			
	60~90	6.5	8.1	6.6	6.5			
Cd μg·kg^−1^	0~30	130 a	132 a	133 b	120 a	≤200 ≤ 1000		
	30~60	105 a	121 a	105 a	106 b			
	60~90	94	104	88	105			
Cu /mg·kg^−1^	0~30	24 a	74 a	64 b	70 a	≤35 ≤ 100 ≤ 400		
	30~60	21 a	70 a	66 a	67 b			
	60~90	17	65	63	68			
Cr /mg·kg^−1^	0~30	73 a	22 b	22 b	21 b	≤90 ≤ 250 ≤ 300		
	30~60	71 a	21 a	19 a	20 a			
	60~90	71	20	16	18			
Hg /μg·kg^−1^	0~30	44 a	48 c	56 b	52 b	≤150 ≤ 1000 ≤ 1500		
	30~60	21 a	23 a	20 a	18 a			
	60~90	12	15	17	16			
Zn /mg·kg^−1^	0~30	60 a	60 b	64 ab	54 c	≤100 ≤ 300 ≤ 500		
	30~60	64 a	60 a	64 a	51 b			
	60~90	61	51	58	48			

Note: In the *p* = 0.05 level, same letters stand for no significant difference; different letters stand for the opposite.

**Table 3:** Soil heavy metals contents with different volumes of unconventional water agricultural utilization.

Index	Depth/cm	Local Values	Full-Clear Water	Half-Unconventional Water	Full-Unconventional Water
As /mg·kg^−1^	0~30	8.4	8.8 a	9.3 a	9.8 a
	30~60	8.1	8.0 a	8.3 a	10.0 c
	60~90	6.5	8.1 a	6.8 a	8.2 a
Cd /μg·kg^−1^	0~30	130	105 a	121 a	126 ac
	30~60	105	96 a	132 b	110 c
	60~90	94	94 ac	104 a	90 a
Cu /mg·kg^−1^	0~30	23	16 a	21 a	22 a
	30~60	22	18 a	20 a	25 a
	60~90	19	16 a	18 ab	23 a
Cr /mg·kg^−1^	0~30	73	60 a	69 a	75 ad
	30~60	71	63 b	69 ab	76 a
	60~90	72	58 a	61 a	65 a
Hg /μg·kg^−1^	0~30	44	51 a	50 a	51 a
	30~60	21	34 a	22 a	25 a
	60~90	12	18 a	15 a	19 a
Pb /mg·kg^−1^	0~30	60	14 a	18 a	22 a
	30~60	64	17 a	18 a	21 a
	60~90	61	14 a	15 a	19 a
Zn /mg·kg^−1^	0~30	7.9	47 a	56 a	60 ab
	30~60	8.1	43 b	52 b	61 b
	60~90	6.8	46 a	48 a	53 a

Note: For the *p* = 0.05 level, same letter means there is no significant difference, and different letters mean there are apparent differences.

**Table 4:** Cucumber fruits heavy metals contents in condition of different irrigation water quality. mg/kg.

Water Quality	As	Cd	Hg	Cr	Cu	Pb	Zn
Clear water	0.0011	0.018	0.0038	0.049	0.014	0.055	No detected
Half-unconventional water	0.0014	0.027	0.0031	0.051	0.015	0.068	No detected
Full-unconventional water	0.0021	0.040	0.0040	0.082	0.0021	0.055	No detected
National standard	0.5	0.05	0.01	0.5	0.5	0.2	No detected

**Table 5:** During the cucumber growth season, changes of heavy metals in soil before and after cucumber harvest. mg/kg.

Factor	Treatment	As	Cd	Cu	Cr	Hg	Pb	Zn
Heavy metals take-away by the aboveground part of cucumber	Full-clear water	61.2514	0.8969	139.9315	48.8856	0.5621	42.0766	310.4012
	Half-unconventional water	45.2648	0.8195	84.8656	46.4768	0.6394	46.8865	289.1585
	Full-unconventional water	54.7521	0.8994	90.7238	58.6648	0.6011	49.6669	301.7634
Heavy metals bring-in with the unconventional water for irrigation	Full-clear water	2.7862	0.0501	3.6645	16.5635	0.0512	1.1084	16. 1066
	Half-unconventional water	3.9120	0.0549	16.1052	24.8666	0.0416	1.7143	130.9985
	Full-unconventional water	5.4268	0.0601	28.8649	30.9963	0.0426	1.6016	250.1006

**Table 6:** During the cucumber growth season, proportion of changes in the amounts of heavy metals in soil before and after cucumber harvest %.

Factor	Treatment	As	Cd	Cu	Cr	Hg	Pb	Zn
Heavy metals take-away proportions account for total volume in soil	Full-clear water	0.6154	0.6928	0.5862	0.0679	0.6298	0.1812	0.4266
	Half-unconventional water	0.5082	0.6102	0.3863	0.0524	0.6110	0.2011	0.4189
	Full-unconventional water	0.6826	0.6926	0.3546	0.0699	0.5986	0.2103	0.3968
Heavy metals bring-in proportions account for total volume in soil	Full-clear water	0.02166	0.0405	0.0103	0.0217	0.2198	0.0051	0.0208
	Half-unconventional water	0.04135	0.0411	0.0663	0.0268	0.1256	0.0060	0.1936
	Full-unconventional water	0.05716	0.0521	0.1256	0.0398	0.0986	0.0061	0.3076

## Data Availability

Not applicable.
